# Predicting outcomes of port-wine stains treated with hematoporphyrin monomethyl ether photodynamic therapy (HMME-PDT): An observational study using multimodal deep learning

**DOI:** 10.1016/j.jdin.2025.11.009

**Published:** 2025-11-21

**Authors:** Jinjin Zhu, Chunhui Xie, Yan Li, Xue Wang, Ziqiong Cheng, Yunqi Li, Juan Tao

**Affiliations:** a Department of Dermatology, Union Hospital, Tongji Medical College, Huazhong University of Science and Technology, Wuhan, China; b Department of Polymer Materials and Engineering, College of Materials and Metallurgy, Guizhou University, Guiyang, China; c Dermatology Department, The First Affiliated Hospital of Shihezi University, Xinjiang, China

**Keywords:** artificial intelligence, hemoporfin-mediated photodynamic therapy, machine learning, multimodal deep learning, port-wine stains, prediction model, vascular malformation, hematoporphyrin monomethyl ether

*To the Editor:* Port-wine stains are common congenital vascular malformations.^1^ Hematoporphyrin monomethyl ether mediated photodynamic therapy (HMME-PDT) has been shown to be an efficacious and safe treatment option.^2^ Nevertheless, its therapeutic outcomes vary substantially with individuals and are difficult to predict, highlighting an urgent need for a reliable predictive tool.

Clinical factors (age, lesion location/type) influence prognosis, but prediction remains challenging.^2^ Our prior work found dermoscopic vascular patterns strongly correlate with HMME-PDT response,^3^ suggesting combining clinical and dermoscopic data could improve prediction. Deep learning enables such integration.^4^^,^^5^

In this study, we developed a multimodal deep learning model to predict HMME-PDT outcomes based on clinical data and dermoscopic images. A total of 239 patients from the Department of Dermatology, Union Hospital, Tongji Medical College, Huazhong University of Science and Technology were included. Eight clinical risk factors—lesion size, location, type (pink/red, purple, thick), color uniformity, texture changes, treatment history, immediate postoperative reaction, and side effects—were combined with dermoscopic images, generating 878 data points. Patients were classified as cured, significantly improved, improved, or ineffective. Data were randomly split into training (80%) and test (20%) sets. Fig 1 illustrates the architecture of the multimodal model, which used a Convolutional Neural Network for dermoscopic image features and a multilayer perceptron (MLP) for clinical factors; extracted features were concatenated and fed into an MLP for classification.

**Fig 1:**
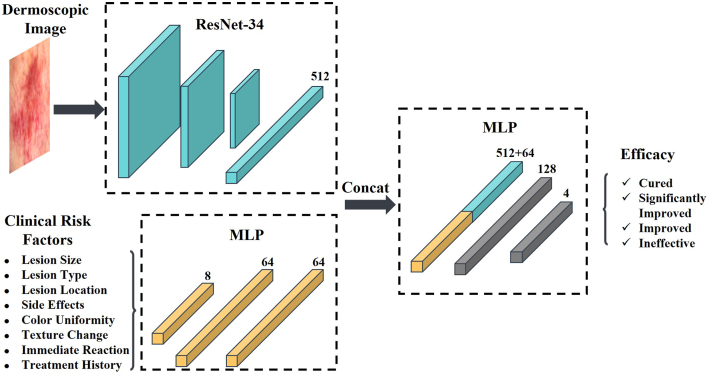
Schematic representation of the architecture of the multimodal deep learning model. ResNet-34^5^ was used as the feature extractor for dermoscopic images, while a multilayer perceptron (MLP) was used to process the clinical risk factors. The features extracted from both modalities were subsequently concatenated and fed into an MLP to predict the outcomes of HMME-PDT. *HMME-PDT*, Hemoporfin-mediated photodynamic therapy.

As illustrated in Fig 2, *A*, the model exhibited robust predictive performance, achieving an overall accuracy of 86.9% on the test dataset, as confirmed by internal testing. The sensitivity for predicting the outcomes of cure, significant improvement, improvement, and ineffectiveness was 78.4%, 92.0%, 87.5%, and 81.3%, respectively, while the corresponding specificities were 97.1%, 89.1%, 95.3%, and 98.8%. These performance metrics closely align with clinical observations, supporting the conclusion that integrating clinical and dermoscopic data enables effective assessment of the relationship between HMME-PDT efficacy and patient-specific characteristics.

**Fig 2:**
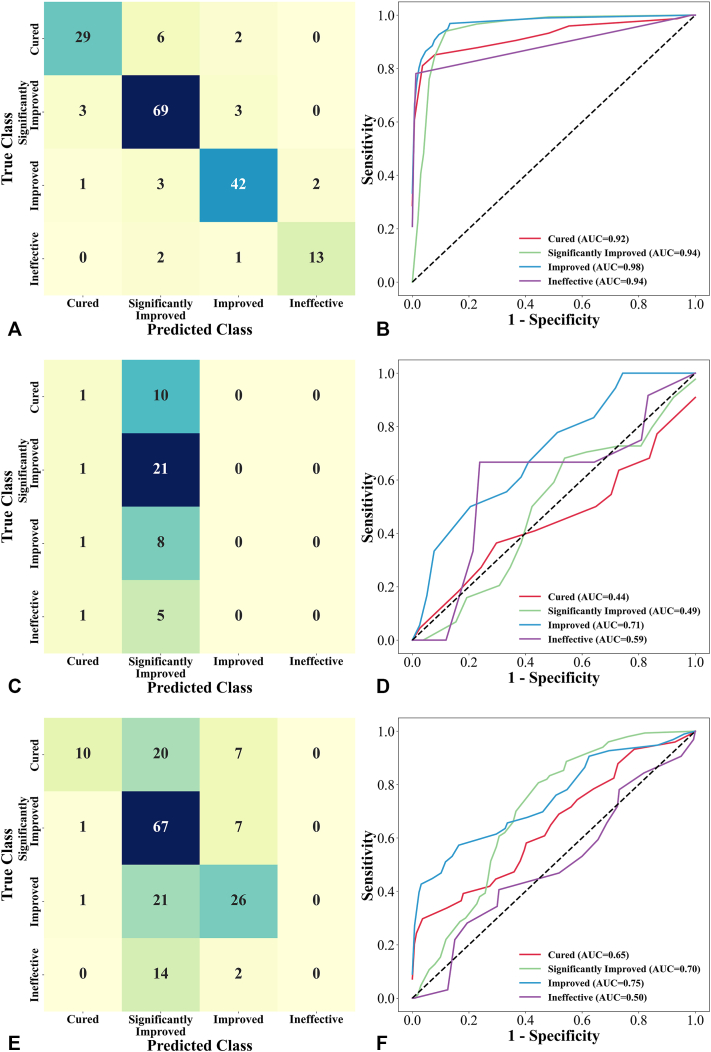
The confusion matrix and receiver operating characteristic (ROC) curve for models using both clinical data and dermoscopic images (**A** and **B**; via the multimodal deep learning model), only clinical data (**C** and **D**; via multilayer perceptron, MLP), and only dermoscopic images (**E** and **F**; via ResNet-34), applied to the test dataset.

To further assess the benefit of multimodal integration, we compared our model with unimodal baselines (Fig 2). Using only clinical data (MLP) achieved 45.83% accuracy (Fig 2, *C*), and only dermoscopic images (ResNet-34) improved accuracy to 58.5% (Fig 2, *E*), indicating greater prognostic value for image-based data but still much lower than the 86.9% achieved by the multimodal model. Receiver operating characteristic curve analysis further confirmed the advantage, showing consistently higher areas under the curve for all outcome categories—cured, significantly improved, improved, and ineffective—in the multimodal approach compared with unimodal models (Fig 2, *B, D, F*). These results demonstrate that integrating clinical and dermoscopic modalities substantially improve predictive accuracy, robustness, and discriminative ability across all classes.

This study demonstrates that multimodal deep learning can effectively integrate clinical and dermoscopic data to predict HMME-PDT outcomes for port-wine stains, offering an early prognostic tool for personalized treatment planning. While preliminary results are promising, the limited sample size constrains generalizability. Future work should focus on larger patient cohorts and additional clinical factors to enhance performance. With expanded datasets, predictive models could achieve greater robustness, better support clinical decision-making, and more accurately manage patient expectations.

## Conflicts of interest

None disclosed.
